# Black Eschars in the Highlands of Ethiopia

**DOI:** 10.4269/ajtmh.15-0763

**Published:** 2016-10-05

**Authors:** Ramón Pérez-Tanoira, Juan Cuadros, Laura Prieto-Pérez

**Affiliations:** 1 Department of Medicine and Laboratory, Gambo Rural General Hospital, Kore, West-Arsi, Gambo, Ethiopia; 2 Department of Otorhinolaryngology-Head and Neck Surgery, University of Helsinki and Helsinki University Hospital, Helsinki, Finland; 3 Department of Microbiology, University Hospital Príncipe de Asturias, Madrid, Spain; 4 Division of Infectious Diseases, IIS-Fundación Jiménez Díaz, Madrid, Spain; 5 Department of Medicine, Universidad Autónoma de Madrid, Madrid, Spain

**Figure 1.**
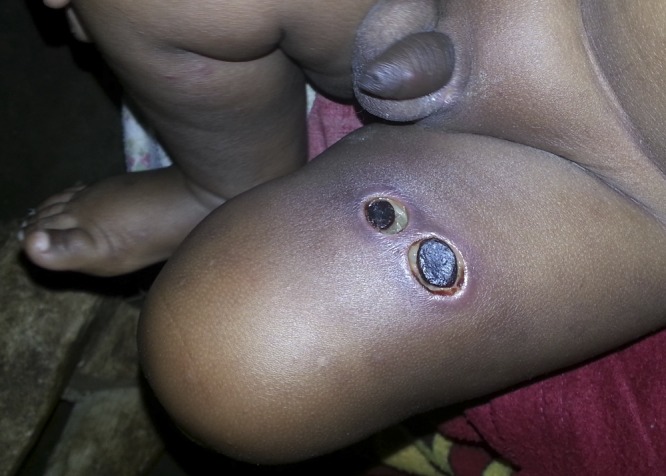
Erythematous and edematous lesions over left thigh taken at the moment of sample collection.

**Figure 2.**
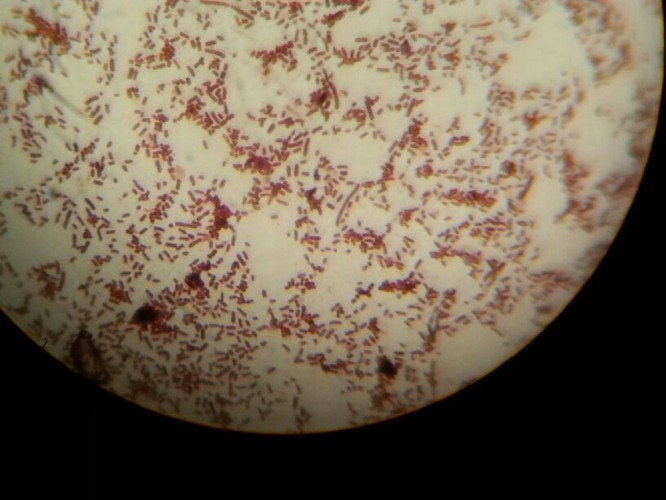
Gram-positive bacilli observed under oil-immersion microscope (× 1000) in a specimen taken from the exudate beneath the eschar.

A 1-year-old boy, living in close contact with farm animals in the rural highlands of southwest Ethiopia, was brought to the emergency room because of two black eschars on his left thigh (Figure 1

) and a painful ipsilateral adenopathy in the groin. According to the clinical history obtained by his mother, the lesions started as tiny papules 4–5 days earlier and had grown to the size of a coin. No other symptoms or signs were found in the clinical examination, and the white blood cell count was 7,600/mm^3^. The physician on call suspected cutaneous anthrax, and a microbiologist obtained a specimen of the exudate just under the eschar. Gram-positive bacilli were seen under the microscope (Figure 2

), and the following day, a grey and dry colony described as “*Bacillus*-like” was seen on the surface of the culture plate.^1^ With the clinical diagnosis of a malignant pustule, the patient was admitted to the hospital and was treated with intravenous penicillin G for 10 days, and the case had a favorable outcome.

Cutaneous anthrax, a disease associated with biological terrorism in western countries, is common and underreported in the rural areas of Africa. It can be lethal in some cases, especially when the oropharyngeal area is affected after ingesting meat from contaminated sources.^2^,^3^ Frequently, the infection can be traced back to contact with a specific, diseased grazing herbivorous animal, and it is seen to occur within families.^4^,^5^ The diagnosis is usually based on the clinical aspect of the lesions, although it can be confused with ecthyma (caused by *Streptococcus pyogenes*, *Staphylococcal aureus*, or other Gram-positive cocci); cutaneous leishmaniasis (*Leishmania major* or *Leishmania tropica*; intracellular amastigotes, observed after Giemsa stain); or other rare infections associated with black eschars, like scrub typhus (*Orientia tsutsugamushi*; an intracellular Gram-negative coccobacillus), rat bite fever, tularemia (*Francisella tularensis*; a Gram-negative coccobacillus), or brown recluse spider (*Loxosceles reclusa*; sphingomyelinase which causes severe necrosis at the bite site).^6^
